# Efficient and Sustainable Bidentate Amines-Functionalized Resins for Removing Ag^+^, Cu^2+^, Pb^2+,^ and Fe^3+^ from Water

**DOI:** 10.3390/polym15132778

**Published:** 2023-06-22

**Authors:** Ana-Laura Villa-Reyna, Milagros Aguilar-Martínez, Adrián Ochoa-Terán, Hisila Santacruz-Ortega, Mario-Alberto Leyva-Peralta, Judas-Tadeo Vargas-Durazo, Moisés I. Salazar-Gastelum, José García-Elías, Juan-Carlos Gálvez-Ruiz

**Affiliations:** 1 Departamento de Ciencias Químico Biológicas y Agropecuarias, Universidad de Sonora, Avenida Universidad e Irigoyen S/N, Col. E. Ortiz, Caborca 83600, Mexico; 2 Tecnológico Nacional de México/Instituto Tecnológico de Tijuana, Blvd. Alberto Limón Padilla S/N, Otay Tecnológico, Tijuana 22510, Mexico; ochoa@tectijuana.mx (A.O.-T.);; 3 Departamento de Investigación Polímeros y Materiales, Universidad de Sonora, Blvd. Luis Encinas y Rosales S/N, Col. Centro, Hermosillo 83000, Mexico; 4 Departamento de Ciencias Químico Biológicas, Universidad de Sonora, Blvd. Luis Encinas y Rosales S/N, Col. Centro, Hermosillo 83000, Mexico

**Keywords:** chelating resin, metals, bioaccumulation, dosimeters, Merrifield resin

## Abstract

We evaluate the effectiveness of chelating resins (CR) derived from Merrifield resin (MR) and 1,2-phenylenediamine (PDA), 2,2’-dipyridylamine (DPA), and 2-(aminomethyl)pyridine (AMP) as adsorbent dosimeters for Ag^+^, Cu^2+^, Fe^3+^, and Pb^2+^ cations from water under competitive and noncompetitive conditions. MR-PDA, MR-DPA, and MR-AMP were obtained in a 95–97% yield and characterized by IR, fluorescence, and SEM. The ability of CRs as adsorbents was determined by batch and flow procedures. MR-PDA showed a batch adsorption capacity order of Fe^3+^ (29.8 mg/g) > Ag^+^ (2.7 mg/g) > Pb^2+^ (2.6 mg/g) at pH 3.4. The flow adsorption showed affinity towards the Ag^+^ cation at pH 7 (18.4 mg/g) and a reusability of 10 cycles. In MR-DPA, the batch adsorption capacity order was Ag^+^ (9.1 mg/g) > Pb^2+^ (8.2 mg/g) > Cu^2+^ (3.5 mg/g) at pH 5. The flow adsorption showed affinity to the Cu^2+^ cation at pH 5 (2.2 mg/g) and a reuse of five cycles. In MR-AMP, the batch adsorption capacity was Ag^+^ (17.1 mg/g) at pH 3.4. The flow adsorption showed affinity to the Fe^3+^ cation at pH 2 (4.3 mg/g) and a reuse of three cycles. The three synthesized and reusable CRs have potential as adsorbents for Ag^+^, Cu^2+^, Fe^3+^, and Pb^2+^ cations and showed versatility in metal removal for water treatment.

## 1. Introduction

Metals are highly dense elements with low electronegativity and are primarily present as solids at ambient temperature [1,2]. A subcategory of metals known as “potentially toxic elements” (PTEs) can lead to toxic outcomes due to bioaccumulation [3]. Silver, zinc, copper, iron, mercury, chromium, and lead are classified as PTEs. Lead is the most toxic and represents a high public health risk because concentrations as low as 1 ppm can be lethal, according to the World Health Organization (WHO) [4]. The abundance of PTEs in the environment shows a relationship with anthropogenic activities—mainly, inadequate toxic waste management from mining [3] or accidental release. These environmental disasters include the 2014 Sonora River, 2019 Sea of Cortes, and 2021 River Tula incidents [3,5,6,7]. These events and the accelerated development of mining activities have been associated with increased cancer cases in the State of Sonora, México. As a result, Sonora has the highest cancer mortality rate in México [3,8,9,10,11,12]. The correlation between exposure to metals and cancer incidence has been studied. For instance, geographical areas with high lead levels have high mortality rates for esophageal and breast cancers. Moreover, high copper concentrations in water, air, and soil increase the incidence of lung cancer [13,14,15,16]. These situations show the adverse impact of PTEs on the environment and public health.

As mentioned earlier, it is crucial to create improved systems for detecting PTEs in water, monitoring their levels, and removing them when they exceed permissible limits. The development of such systems is of utmost importance. However, eliminating PTEs is complex because of their toxicity, even at low concentrations. These substances are typically present in intricate mixtures with organic or inorganic materials. Various methods have been tested to remove PTEs from water, which have some advantages and limitations, such as the case of inefficient chemical precipitation when PTEs concentrations are low and are a high-cost procedure. In the same way, it happens with membrane filtration, flotation, and the use of electrochemical techniques, which, in addition to being expensive, can generate a large amount of waste. Therefore, Ihsanullah A et al. (2017) critically reviewed these methods [17]. One of the preferred procedures for removing PTEs is adsorption because of its low cost, ease of handling, and advanced generation of new and efficient sorbent materials [18].

Some materials that can be useful for sensing and removing PTE are chelating resins (CRs). CRs are copolymers composed of a polymer support that usually bears a polydentate molecule that can chelate metal ions. A CR is called a sensor when it provides a helpful signal in response to a particular analyte’s presence [19,20]. In addition, the resin should also exhibit sensitivity, selectivity, stability, a fast response time, environmental friendliness, and reusability. Materials that meet the abovementioned criteria are called dosimeters [21]. Unfortunately, some CRs fail to meet all these characteristics—mainly, selectivity (similar affinity to various metals), sensitivity (quickly saturated or blocked), reusability (cost and time for regeneration), and reproducibility (decrement in sensitivity after adsorption–desorption cycles). 

When searching for effective CRs for removing PTEs, it is crucial to consider the impact of both the polymeric matrix and the chelating unit. This consideration is essential for creating a material with specific properties. Merrifield resin is the most commonly used solid support for synthesizing peptides and CR since it has good thermomechanical stability and allows for the recovery and reuse of the CR [21,22,23,24,25]. In this context, Pina-Luis et al. (2009) reported the synthesis of some CRs derived from Merrifield resin and amino benzamides labeled with a fluorescent group that showed high sensitivity to magnesium (Mg^2+^) [24]. By 2012, they reported the synthesis of a CR based on Merrifield resin and morin. This resin showed a high retention capacity for Cu^2+^ and Pb^2+^ (95% for both ions) at pH 7 [25].

In 2010, Ghassabzadeh et al. reported using expanded perlite to remove Ag^+^, Cu^2+^, and Hg^2+^ ions from aqueous media at an optimal pH of 6.5. The absorption capacities of Ag^+^, Cu^2+^, and Hg^2+^ by perlite were 8.45, 1.95, and 0.35 mg/g, respectively, showing a higher absorption of Ag^+^ ions [26]. Martinez-Meza (2017) prepared the CR Dowex-M4195a with the ability to absorb Fe^3+^ cations. This resin was mentioned as an alternative to conventional treatments for removing PTEs from an aqueous solution [27].

In 2021, Zou et al. prepared a CR based on a polychloromethylestyrene resin containing heterfluorenone pendant groups to remove heavy metals such as Cu^2+^, Pb^2+^, and Ni^2+^. This CR is a promising adsorbent of metal ions at pH 5, showing maximum adsorption capacities of 0.16, 0.28, and 0.55 mg/g of Cu^2+^, Pb^2+^, and Ni^2+^, respectively [28]. Furthermore, Duan et al. (2022) reported that, at pH 5, the metal ions Cu^2+^ and Pb^2+^ were adsorbed by a poly(6-acryloylamino-*N*-hydroxyhexanamide) resin, showing an adsorption capacity of 238.59 and 232.48 mg/g, respectively [29]. 

Previously, we used solid-phase organic synthesis due to its inherent advantages for developing efficient dosimeters with a high potential for metal detection, including easy purification steps, high yields, and fast reaction rates. We prepared eight CRs from Merrifield resin and bidentate amines, obtaining potential dosimeters for alkaline cations such as sodium [23]. Now, we want to contribute to advancing the field by synthesizing three CRs derived from Merrifield resin and bidentate amines and determining their potential as reusable chemical dosimeters for removing Ag^+^, Cu^2+^, Fe^3+^, and Pb^2+^ cations from water. The CRs synthesized in this work possess unique properties that make them potentially effective in eliminating PTEs. The geometry of the pre-organized chelating unit promotes the formation of stable chelates of five- or six-member rings, ensuring the stability of the complex formed. Furthermore, the weakly basic secondary and aromatic amines in the new resins make them suitable for a wide pH range (2–7) commonly found in waste materials. At the same time, the varying levels of localized lone pair electrons in the nitrogen atoms may aid in forming stable chelates for removing metals while being labile enough to allow the CR to be reused. The rigidity of the PDA, the intermediate flexibility of the AMP, and the presence of three nitrogen atoms in the DPA may improve the sorption properties of these CRs. It is worth noting that these resins demonstrate versatility and functionality in this regard

## 2. Materials and Methods

Merrifield resin and amines were commercially obtained from Sigma Aldrich (Mexico City, Mexico). The resin had a chlorine loading of 4.5 mmol/g, a crosslinking of 2% divinylbenzene, and a 100–200 mesh particle size. Methanol, diethyl ether, DMF, and THF (Sigma Aldrich) were used as solvents without pretreatment. Deionized water was purified using a Thermo Scientific Barnstead Smart2Pure system. Metallic salts (AgNO_3_, Cu(NO_3_)_2_, Fe(NO_3_)_3_, Pb(NO_3_)_2_) and K_2_CO_3_ were purchased from Sigma Aldrich and used without further purification.

Infrared (IR) spectra were obtained using a Perkin Elmer/ATR Frontier spectrometer. Fluorescence analysis was performed on a Shimadzu RF spectrofluorimeter using a solid sample and flow cells. UV-Vis spectra were collected with a Genesys 10 UV-Vis spectrophotometer. A high-resolution dispersive X-ray scanning electronic microscope (SEM, JEOL) was used for imaging. 

The Perkin Elmer Optima 8300 spectrometer was used to perform the ICP-OES analysis. The Ar plasma gas, auxiliary, nebulizer, and sample flow rates were adjusted to 15.00 L min^−1^, 0.20 L min^−1^, 0.55 L min^−1^, and 1.5 mL min^−1^, respectively. The analysis was conducted using a radio frequency of 1300 W in the axial mode. The wavelengths utilized to determine the quantities of Ag, Cu, Fe, and Pb were 328 nm, 327 nm, 238 nm, and 220 nm, respectively. All experiments were conducted in triplicate.

### 2.1. Synthesis and Characterization of Chelating Resins

Merrifield resin (0.9 mmol) and DMF (15 mL) were poured into a 20 mL vial. Then, 2.77 mmol of the corresponding amine and 3.6 mmol of K_2_CO_3_ were added, and the reaction mixture was constantly swirled for four hours using an orbital shaker (VWR Mini Shaker). Afterward, the mixture was vacuum-filtered and rinsed three times using 30 mL of water, methanol, ethyl ether, and THF on every rinse. Finally, the synthesized CR was oven-dried for 12 h at 40 °C. After repeating this procedure three times, all the reactive sites were substituted. The products were characterized by IR spectroscopy, fluorescence spectroscopy, and the Volhard method [23,26,27,28].

MR-PDA: 95%; conversion 99%, IR: chelating amine 3349, 3307, 1580, 1209; matrix, 3018, 2923, 1594, 1435, 744, 689 cm^−1^; fluorescence: λ_em_ = 440 nm (λ_ex_ = 382 nm). 

MR-DPA: 98%; conversion 98%; IR: chelating amine 1181; matrix, 3018, 2917, 1495, 1428, 737, 689 cm^−1^; fluorescence: λ_em_= 465 nm (λ_ex_ = 408 nm). 

MR-AMP: 98%; conversion 98%; IR: chelating amine, 3326, 1071; matrix, 3023, 2917, 1422, 749, 689 cm^−1^, fluorescence: λ_em_= 439 nm (λ_ex_ = 384 nm). 

### 2.2. Methods for the Evaluation of Sensing Properties of Chelation Resins

Batch Method for the Selectivity Analysis of Chelating Resins. We performed competition studies by dissolving 2.7 mmol cation salt (1:1 ratio) in a vial with 15 mL of deionized water. Then, the corresponding CR (0.9 mmol) was added, and the mixture was swirled for six hours, followed by filtering, rinsing, and oven-drying for 12 h at 40 °C. Then, the solids were analyzed by fluorescence spectroscopy, MR-PDA: λ_ex_ 382 nm, λ_em_ 439 nm; MR-DPA: λ_ex_ 420 nm, λ_em_ 481 nm; MR-AMP: λ_ex_ 342 nm, λ_em_ 408 nm.

Flow Method for the Selectivity Analysis of Chelating Resins. Aqueous solutions were prepared using a single cation or combinations thereof at a final concentration of 10 mM. Next, the CR (0.1 g) was packed into a fluorescence flow cell. Finally, the aqueous solution to be tested was passed through the flow cell using a peristaltic pump, maintaining a constant flow of 2 mL/min. Automated fluorescence measurements were performed every 2 min for 82 min (MR-PDA: λ_ex_ 382 nm, λ_em_ 439 nm; MR-DPA: λ_ex_ 420 nm, λ_em_ 481 nm; MR-AMP: λ_ex_ 342 nm, λ_em_ 408 nm).

Flow Method for the Saturation Analysis of Chelating Resins. Each CR (0.1 g) was packed in the flow cell, and the cation solution (10 mM concentration) was passed through the flow cell at 2 mL/min for 180 min with a peristaltic pump. Fluorescence measurements were performed every 5 min until CR saturation (MR-PDA: λ_ex_ 388 nm, λ_em_ 442 nm; MR-DPA: λ_ex_ 381 nm, λ_em_ 420 nm; MR-AMP: λ_ex_ 347 nm, λ_em_ 402 nm).

#### 2.2.1. Tests for the Evaluation of the Adsorption Properties of Chelating Resins

Column test: Solutions of each cation were prepared at 10 ppm with a pH range between 2 and 7. In total, 0.1 g of the resin was packed in a glass column of 6 cm in length. Using a peristaltic pump, a total volume of 20 mL of the solutions of the corresponding cation was passed at a constant flow of 10 mL/min. Both the initial and residual solutions were analyzed by Uv-Vis spectroscopy (382 nm Ag^+^, 510 nm Cu^2+^, 240 nm Fe^3+^).

Batch test: In total, 10 mL of a solution containing a mixture or individual cations were placed into a 20 mL vial. Then, the CRs MR-PDA, MR-DPA, and MR-AMP and the control resin (Merrifield resin) were added to the vials in different amounts (10, 20, 30, 40, 50, 75, 100, and 150 mg). Then, the mixture was put in orbital shaking for six hours at room temperature. Next, the pH was adjusted by adding a NaOH-concentrated solution (5 for MR-DPA and 3.4 for MR-PDA and MR-AMP). Finally, the resin was removed by filtration. 

The supernatant was evaluated to determine the residual concentration of the metal using the ICP-OES atomic emission technique. This experiment was performed in triplicate, and the final concentration values correspond to the average of the three measurements. 

The retention percentage, *R* (%), was calculated using Equation (1):

(1) $$R\left(\%\right)=\frac{\left(C_{i}-C_{f}\right)}{C_{i}}\times 100$$

where:

*C_i_* = initial concentration of the metal ion (ppm)

*C_f_* = final concentration of the metal ion (ppm)

The adsorption capacity, *Q_e_* (mg/g) at equilibrium, was calculated from the differences between the metal quantity added to the resins and the metal content of the supernatant, using the following equation:

(2) $$Qe=\frac{\left(C_{i}-C_{e}\right)V}{m}$$

where:

*C_i_* = initial concentration of the metal ion (mg/L)

*C_e_* = concentration of the metal ion in the supernatant (mg/L) at equilibrium

*V =* volume of the metal ion solution (L)

*m* = mass of the resin in grams

#### 2.2.2. Chelating Resin Reusability Test

A similar packed column test was performed to determine for how many cycles the CRs could be employed. The experiments were performed at the resulting pH of the solution (4.3 for Ag^+^, 5.0 for Cu^2+^, and 2.4 for Fe^3+^). First, a 20 mL cation solution (10 ppm, 10 mL/min) was passed through the packed column. Then, 5 mL of 5% nitric acid was added, followed by 20 mL of deionized water to release the retained cation from the resin. This procedure was repeated ten times under the same conditions. Finally, the initial and residual solutions were analyzed by UV-Vis spectroscopy (382 nm Ag^+^, 510 nm Cu^2+^, 240 nm Fe^3+^). The CRs were kept and used in a new adsorption–desorption cycle.

## 3. Results and Discussion

### 3.1. Synthesis and Characterization of Chelating Resins 

**Figure 1:**
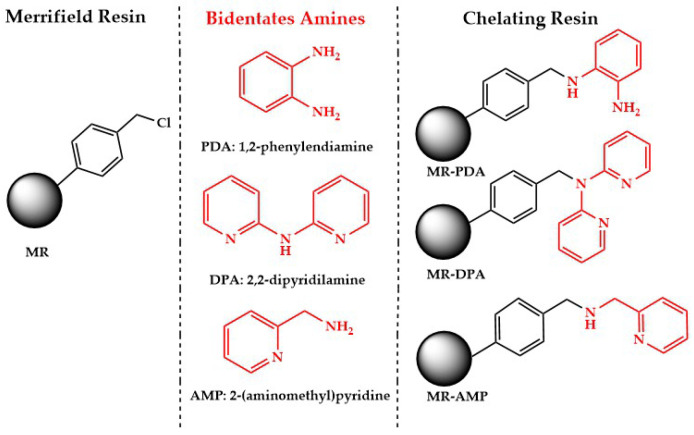
Preparation of CRs. MR-PDA: Merrifield resin-1, 2-phenylendiamine, MR-DPA: Merrifield resin-2, 2’-dipyridilamine, and MR-AMP: Merrifield resin-2-(amino methyl)pyridine.

Three CRs (MR-PDA, MR-DPA, and MR-AMP) derived from Merrifield resin (MR) and 1,2- phenylenediamine (PDA), 2,2’-dipyridylamine (DPA), and 2-(aminomethyl)pyridine (AMP) were obtained with yields between 95 and 97% after repeating the protocol three times (Figure 1).

IR analysis confirmed the support of the different amines on the Merrifield resin. For example, bands corresponding to the amine group were observed, and the absence of the characteristic carbon-chlorine stretching band (around 740 cm^−1^) in the Merrifield resin was noticed. It was observed that MR-PDA displayed bands at 3349 cm^−1^, 3307 cm^−1^, and 1580 cm^−1^, which correspond to the N-H stretching and bending bands, respectively. A band at 1209 cm^−1^ was also noticed, corresponding to the C-N stretching. On the other hand, MR-AMP exhibited single bands at 3326 cm^−1^ and 1071 cm^−1^, which correspond to the N-H and C-N stretchings, respectively. Lastly, MR-DPA displayed only a C-N stretching band at 1181 cm^−1^. The chlorine content was determined by the Volhard method, which showed 100% conversion after the third protocol.

**Figure 2:**
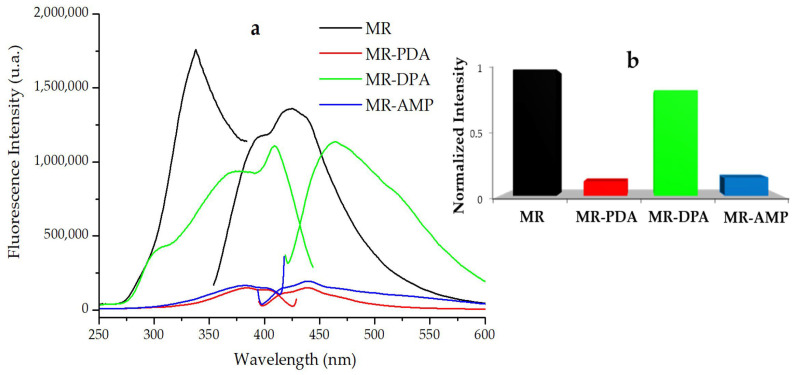
Spectroscopical properties of the synthesized resins. (**a**) Fluorescence emission spectral changes of the Merrifield and CRs. (**b**) Normalized fluorescence emission intensity. MR, Merrifield resin; MR-PDA, Merrifield resin-1,2-phenylenediamine; MR-DPA, Merrifield resin-2,2’-dipyridylamine); MR-AMP, Merrifield resin-2-(aminomethyl)pyridine. Analyses were performed in solid, λ_ex_ = 334 nm.

The support of PDA, DPA, and AMP on the Merrifield resin was evident by observing the fluorescence spectra of the CRs (Figure 2). Resin MR-DPA exhibited the highest fluorescence intensity, while the resins MR-PDA and MR-AMP exhibited a quenching of fluorescence emission of approximately 90%. The behavior in the fluorescence emission shown by MR-PDA and MR-AMP is due to the structural similarity between the binders DPA and AMP. The structural similarity leads to a photoinduced electronic transfer (PET) process generated by the presence of amino groups. The increase in fluorescence intensity observed in MR-DPA is due to the structural rigidity of DPA. The presence of broad and less structured bands of MR-DPA can be attributed to substituted aromatic rings [29,30,31]. A redshift was observed in the fluorescence bands of the CRs compared to the Merrifield resin due to the increase in the number of substituted aromatic rings supporting the amine. MR-DPA showed the most significant band shift at 465 nm.

**Figure 3:**
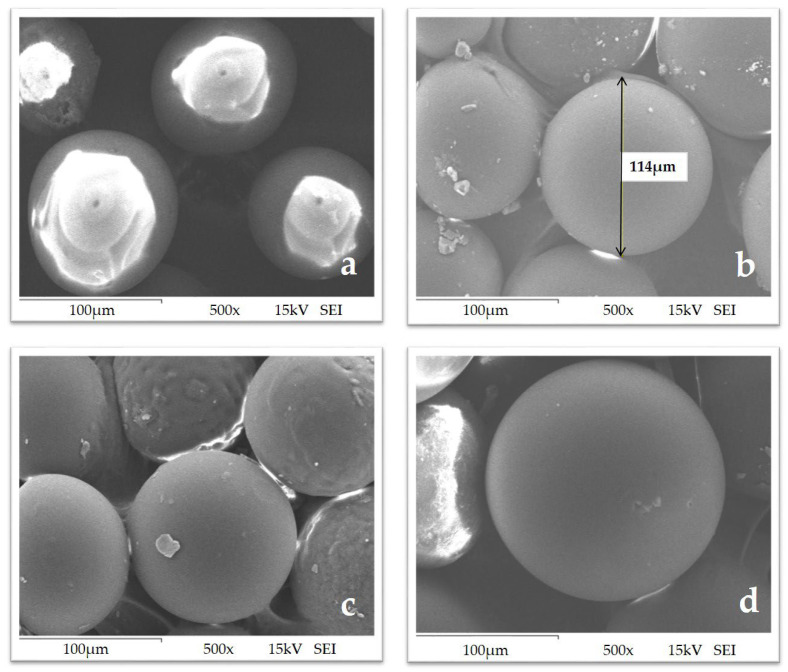
Scanning electron microscope images of (**a**) Merrifield resin, (**b**) MR-DPA: Merrifield resin-2,2’-dipyridylamine, (**c**) MR-DPA: Merrifield resin-2,2’-dipyridylamine, and (**d**) MR-AMP: Merrifield resin-2-(aminomethyl)pyridine. The photos were taken at 15 kV, 500× magnification, and a scale bar of 100 µm.

The micrographs obtained by SEM allowed us to observe the morphology of the resin, and no changes, ruptures, or deformations of the CRs were detected, ensuring the structural integrity of the resin (Figure 3). This technique was used by Islam et al. (2013) to confirm the morphology of the Amberlite XAD-16 CR anchored with glyoxal-bis(2-hydroxy anil), which showed selectivity for aluminum (Al^3+^) [32].

### 3.2. Sensing Properties of Chelating Resins towards Metal Cations

**Figure 4:**
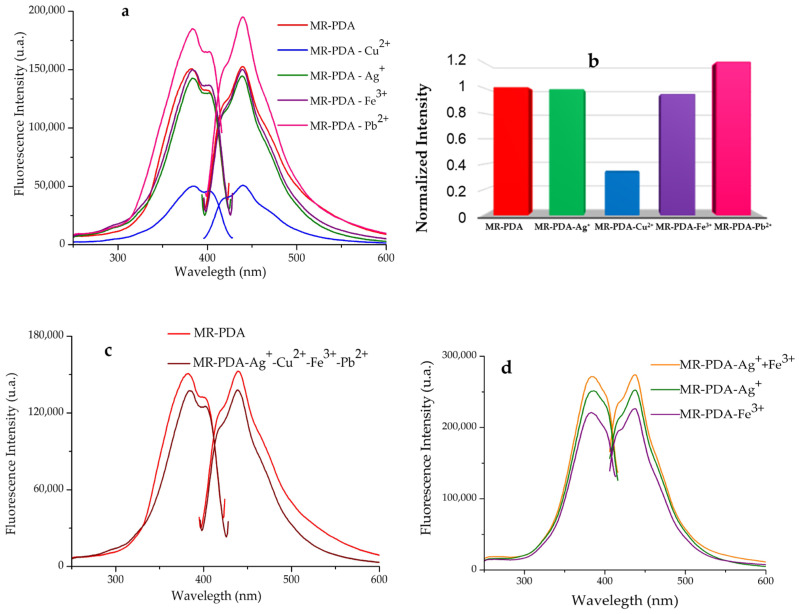
Sensing properties of MR-PDA using the batch method. (**a**) MR-PDA with Cu^2+^, Ag^+^, Fe^3+^, and Pb^2+^ (2.7 mM). (**b**) Normalized intensity. (**c**) MR-PDA vs. MR-PDA with Cu^2+^, Ag^+^, Fe^3+^, and Pb^2+^ (10 mM). (**d**) MR-PDA-Ag^+^ and MR-PDA-Fe^3+^ vs. MR-PDA with Ag^+^ and Fe^3+^ (10 mM). Analyses were performed in solid, λ_ex_ = 334 nm.

To determine the MR-PDA selectivity and affinity for a particular cation in the presence of other cations in the solution [20], we prepared a quaternary mixture of Cu^2+^, Ag^+^, Fe^3+^, and Pb^2+^ cations (each at 10 ppm) to conduct the study. Using the batch method, Figure 4a shows the fluorescence spectra of the solids derived from MR-PDA. The fluorescence spectrum displayed a band with a maximum of 439 nm and an intensity of 137,960 a.u. (Figure 4b). The characteristics of this band resembled those obtained for MR-PDA-Ag^+^ (439 nm; 144,517 a.u.), MR-PDA-Fe^3+^ (439 nm; 150,184 a.u.), and MR-PDA-Pb^2+^ (440 nm, 195,021 a.u.). The results suggest that the presence of Ag^+^, Fe^3+^, and Pb^2+^ cations interferes with the adsorption of Cu^2+^ by MR-PDA.

Therefore, a competition study was performed between Ag^+^ and Fe^3+^ (1:1). Figure 4c shows that the emission band’s peak is at 438 nm and has an intensity of 273,960 a.u. This intensity level is not similar to that of any of the bands produced with MR-PDA-Ag^+^ or MR-PDA-Fe^3+^. This observation indicates the possibility of a synergistic effect caused by the presence of both cations. Based on the fluorescence results, MR-PDA can coordinate Pb^2+^, Ag^+^, and Fe^3+^.

**Figure 5:**
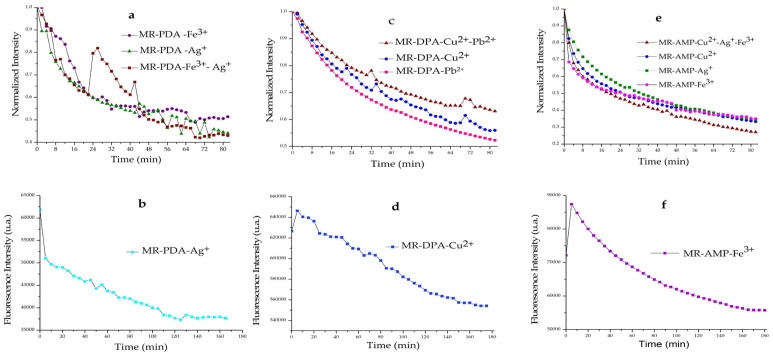
Selectivity analysis of CR for different metals. (**a**,**c**,**e**) Flow method selectivity tests of MR-PDA, MR-DPA, and MR-AMP, respectively. (**b**,**d**,**f**) Saturation tests of MR-PDA-Ag^+^, MR-DPA-Cu^2+^, and MR-AMP-Fe^3+^ (10 mM), respectively. Analyses were performed in the solution at 10 mM, pH 3.4, rate 2 mL/min, MR-PDA, λ_ex_ 382 nm; MR-DPA, λ_ex_ 420 nm; MR-AMP, λ_ex_ 342 nm.

Based on the previous results, a flow method selectivity analysis was carried out for MR-PDA by passing different solutions of the cations Ag^+^, Fe^3+^, and Ag^+^-Fe^3+^ (1:1) through the resin and measuring the fluorescence every 2 min. As shown in the graphic obtained at the end of the analysis, at 82 min, a decrease in fluorescence intensity was observed for the three solutions over time. The fluorescence intensity of the solution with the Fe^3+^ cation decreased by 49%, which was similar to that of the metal cations Ag^+^ and Ag^+^-Fe^3+,^ which fell by 66% (Figure 5a). Those results indicate that MR-PDA may have an affinity for Ag^+^ in the presence of the four metal cations used. Following this result, we performed a saturation test MR-PDA for Ag^+^, which showed saturation after 140 min with a decrease in the fluorescence intensity from 51,000 a.u. to 37,900 a.u. (Figure 5b).

**Figure 6:**
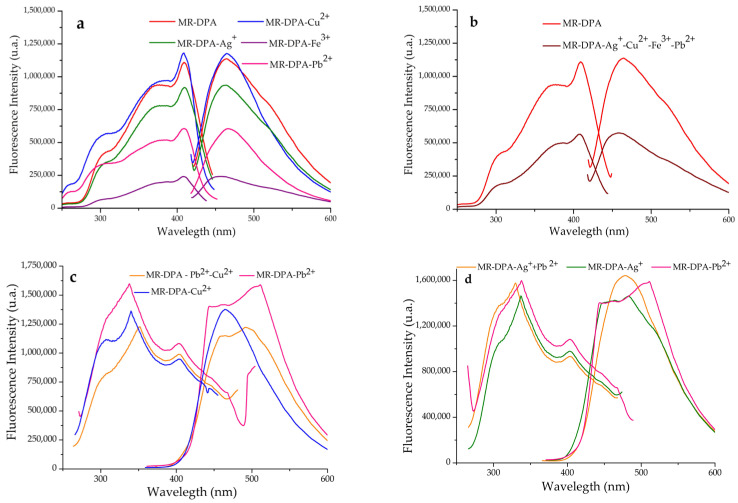
Sensing properties of MR-DPA using the batch method. (**a**) MR-DPA with Cu^2+^, Ag^+^, Fe^3+,^ and Pb^2+^ (2.7 mM). (**b**) MR-DPA vs. MR-PDA with a mixture of Cu^2+^, Ag^+^, Fe^3+,^ and Pb^2+^ (10 mM). (**c**) MR-DPA-Pb^2+^ and MR-DPA-Cu^2+^ vs. MR-DPA with a mixture of Pb^2+^ and Cu^2+^ (10 mM). (**d**) MR-DPA-Pb^2+^ and MR-DPA-Ag^+^ vs. MR-DPA with a mixture of Pb^2+^ and Ag^+^ (10 mM). Analyses were performed in solid, λ_ex_ = 334 nm.

When testing the fluorescence spectra of MR-DPA with various cations, it was observed that the fluorescence intensity decreased with the Ag^+^, Pb^2+^, and Fe^3+^ cations but not with Cu^2+^ (Figure 6a). The MR-DPA-Fe^3+^ complex displayed a unique behavior because its fluorescence intensity decreased by over 80%. Subsequently, a quaternary mixture of the Cu^2+^, Ag^+^, Fe^3+^, and Pb^2+^ cations (each at 10 ppm) was used to conduct the study. The fluorescence spectrum corresponding to this study displayed a band with a maximum of 458 nm and an intensity of 574,631 a.u. (Figure 6b). The characteristics of this band were similar to those obtained for MR-DPA-Pb^2+^ (466 nm; 604,720 a.u.). This CR had selectivity for Pb^2+^ in the presence of Ag^+^, Cu^2+,^ and Fe^3+^ cations.

The first experiment corresponding to the competition studies of MR-DPA involved a binary mixture of Pb^2+^ and Ag^+^ (1:1). MR-DPA displayed an affinity for Pb^2+^ (478 nm; 1,642,151 a.u.), as shown in Figure 6d. The second experiment was conducted in a binary mixture of Pb^2+^ and Cu^2+^ (1:1). The results indicated that MR-DPA showed an affinity for Cu^2+^, as seen in the spectrum, which showed a band (492 nm; 1,221,603 a.u.) very similar to the MR-DPA-Cu^2+^ band (465 nm; 1,376,094 a.u., Figure 6c). Therefore, MR-DPA showed an affinity for both Cu^2+^ and Pb^2+^.

It is shown in Figure 5c that MR-DPA displays selectivity for Cu^2+^. This was also supported by the saturation time observed at 170 min and the decrease in the fluorescence intensity from 646,229 a.u. to 554,129 a.u., as shown in Figure 5d. 

**Figure 7:**
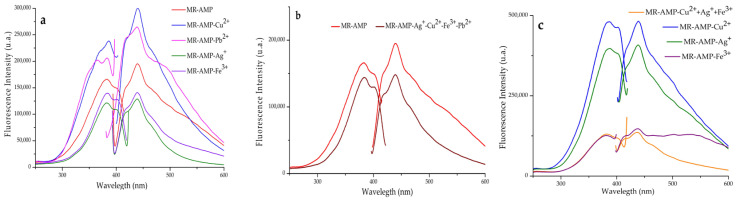
Sensing properties of MR-AMP using the batch method. (**a**) MR-AMP with Cu^2+^, Ag^+^, Fe^3+^, and Pb^2+^ (2.7 mM). (**b**) MR-AMP and MR-AMP with a mixture of Cu^2+^, Ag^+^, Fe^3+,^ and Pb^2+^ (10 mM). (**c**) MR-AMP-Ag^+^, MR-AMP-Fe^3+^ and MR-AMP-Cu^2+^ vs. MR-PDA with a mixture of Cu^2+^- Ag^+^ and Fe^3+^ (10 mM). Analyses were performed in solid, λ_ex_ = 334 nm.

A decrease in fluorescence intensity was observed for the complexes derived from MR-AMP with Fe^3+^ and Ag^+^ (28% and 34%, respectively), and an increase was observed for those with Cu^2+^ and Pb^2+^ (53% and 35%, respectively) (Figure 7a). In the selectivity analysis of the solid complexes, MR-AMP showed an affinity for Fe^3+^ (Figure 7b). This affinity was determined by mixing MR-AMP with a quaternary mixture of Cu^2+^, Ag^+^, Fe^3+^, and Pb^2+^ cations. It was observed that the band corresponding to that mixture resembles that of MR-AMP-Fe^3+^ (439 nm; 148,320 a.u. and 439 nm; 140,842 a.u., respectively; Figure 7a,b). The same behavior was observed when MR-DPA was in the presence of a ternary mixture of Cu^2+^-Ag^+^-Fe^3+^ cations; the band corresponding to this mixture was similar to the band shown in the complex with Fe^3+^ (437 nm; 135,968 a.u. and 438 nm; 147,569 a.u.) (Figure 7c).

MR-AMP’s flow method selectivity test used a ternary mixture of Ag^+^, Fe^3+,^ and Cu^2+^ cations. The spectra of the MR-AMP-Fe^3+^ complex and the corresponding MR-AMP-Cu^2+^-Ag^+^-Fe^3+^ mixture showed more remarkable similarity in the spectral behavior. This could indicate that MR-AMP has a greater affinity for Fe^3+^ in the flow method than in the batch method (Figure 5e). Based on these results, the MR-AMP saturation test was performed in the presence of an Fe^3+^ solution. Figure 5f shows that, at time 165 min, the fluorescence intensity was constant, which indicates that MR-AMP reached maximum coordination with this cation.

Based on the results, each CR exhibited unique behavior during the selectivity tests based on the supported ligands on the resin. The structural variations include the number of aromatic rings in the amine and the metal cations. Fe^3+^, Cu^2+^, and Ag^+^ exhibited an expected decrease in fluorescence intensity. This effect is observed regularly in cation complexes due to the heavy atom effect generated in the molecule [33,34]. This effect increases the probability of intersystem crossing and is responsible for the 30% reduction in fluorescence intensity observed in MR-PDA-Cu^2+^ (Figure 4a). Pina-Luis et al. (2010) observed similar behavior on a CR for detecting Fe^3+^, Cu^2+^, and Zn^2+^. Li et al. (2019) also reported a decrease in fluorescence caused by Cu^2+^ in a natural fluorescent peptide probe with high performance and sensitivity [35].

The high fluorescence intensity observed in the complexes formed by CR and Pb^2+^ comes from the structural rigidity achieved after complexation (Figure 4a). A more rigid structure limits molecular vibrations, which minimizes collision degradation and intersystem crossing [30].

### 3.3. Adsorption Properties of Chelating Resins

**Figure 8:**
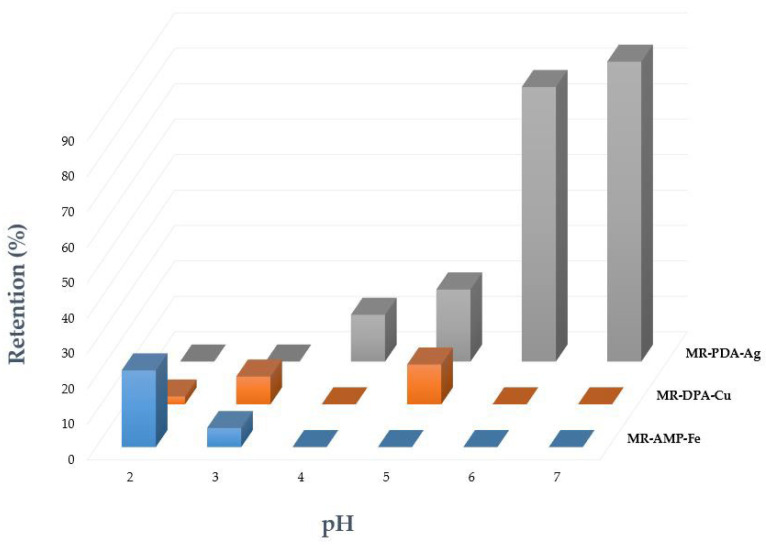
The cation retention percentage of CRs at varying pH values. The blue bar shows the data for the MR-AMP-Fe^3+^, the orange bar shows the data for the MR-DPA-Cu^2+^, and the grey bar shows the data for the MR-PDA-Ag^+^. Analyses were performed at 10 ppm, pH between 2 and 7, rate 10 mL/min, UV-Vis 382 nm, Ag^+^; 510 nm, Cu^2+^; 240 nm, Fe^3+^.

Flow column procedures were subsequently performed to determine the cation adsorption capacity as a function of the pH for each CR and the metal whose selectivity was previously found. Each solution was prepared at the same concentration (10 ppm) at a pH between 2 and 7. For MR-PDA, optimal results were obtained at a 10 mL/min flow rate with an Ag^+^ retention of 78% and 85% at pH 6 and 7, respectively. At pH 5 and 4, the retention values were below 21% at both tested rates. The highest adsorption capacity of MR-PDA was 18.4 mg Ag^+^/g at pH 7 and a 10 mL/min flow rate (Figure 8, Figures S1 and S2 and Table S1).

The adsorption values of MR-DPA were shallow. The most efficient retention of Cu^2+^ was 11% at pH 5, and its adsorption capacity was 2.2 mg Cu^2+^/g (rate = 10 mL/min; see Figure 8). The best conditions for Fe^3+^ retention by MR-AMP were at pH 2 and a 10 mL/min flow rate, resulting in a 22% retention (4.3 mg Fe^3+^ /g) (Figure 8).

A modified batch test was performed to evaluate the removal of metal ions from water using different amounts (10 to 150 mg) of CRs added to 10 mL of an aqueous solution containing a mixture of metal ions at a specific pH. The pH used was similar to that found in water from the improper management of toxic mining waste. After the treatment, the CRs were separated from the solution by filtration, and ICP-OES measured the remaining concentration of metal ions. The adsorption capacity of CR (*Q_e_*, mg/g) for cation removal was calculated according to Equation (2).

**Figure 9:**
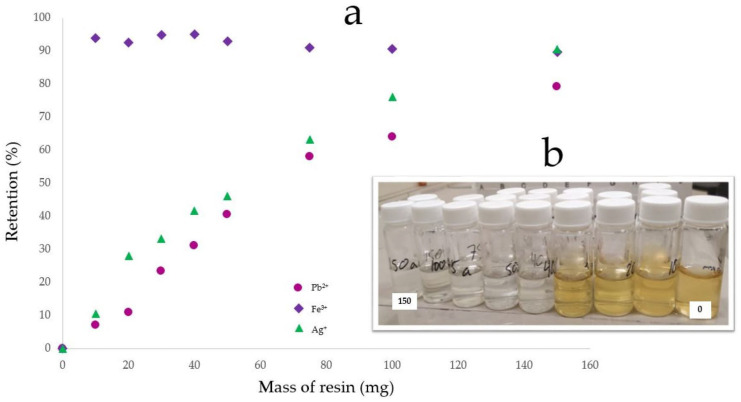
Retention percentage of metals with MR-PDA. (**a**) Effect of the MR-PDA dose on the cation’s retention percentage in a modified batch test. (**b**) Metals solutions at different amounts of MR-PDA, from right to left: 0, 10, 20, 30, 40, 50, 75, 100, and 150 mg. Analyses were performed at 6 h and pH 3.6. Each value is the mean of three independent experiments; standard deviation below 5%.

The MR was evaluated as blank at a 150 mg dose. The resin retained 26% of Pb^2+^ (0.9 mg Pb^2+^/g), 83% of Fe^3+^ (1.8 mg Fe^3+^/g), and 42% of Ag^+^ (1.3 mg Ag^+^/g) without removing Cu^2+^. The cation adsorption capacity with MR-PDA was performed using solutions at pH 3.6 containing Cu^2+^ (46 ppm), Pb^2+^ (49 ppm), Fe^3+^ (32 ppm), and Ag^+^ (45 ppm), which took a yellow color. The results showed that MR-PDA achieves 93% Fe^3+^ removal since 10 mg of resin was added (29.8 mg Fe^3+^/g). This resin also adsorbs 80% of Pb^2+^ (2.6 mg Pb^2+^/g) and 91% of Ag^+^ (2.7 mg Ag^+^/g) at the higher dose of resin (150 mg) but does not adsorb Cu^2+^. Notably, the cations solution changed from yellow to colorless after the treatment with a 40 mg dose of MR-PDA (Figure 9). 

According to the findings, MR adsorbs cations, apparently, by the hydrophobic ion pairing mechanism [36]. The extent of the removal varies depending on the charge and size of the metal ion, as exemplified by Fe^3+^. It has been observed that resins prefer ions at high valence when the solution is low-concentrated. The preference increases inversely to the concentration of the solution [37]. However, further research may be necessary to confirm these observations. It is important to note that the chelating molecule determines the metal’s retention percentage. When 10 mg of MR-PDA is used, 93% of the cation is adsorbed, whereas 150 mg of MR is required to remove 83% Fe^3+^. Additionally, the presence of the PDA significantly affects the adsorption capacity for Pb^2+^ (MR: 26%, MR-PDA: 80%) and Ag^+^ (MR: 42%, MR-PDA: 91%) (Table 1).

**Figure 10:**
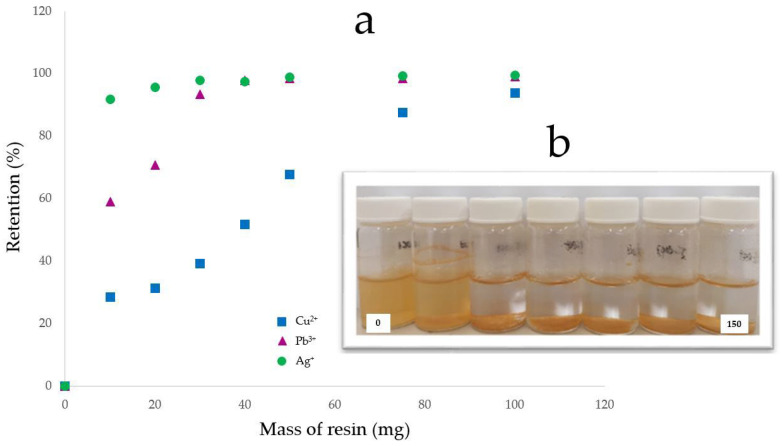
Retention percentage of metals with MR-DPA. (**a**) Effect of the MR-DPA dose on the cation’s retention percentage in a modified batch test. (**b**) Metals cations solutions at different amounts of MR-DPA, from right to left: 0, 10, 20, 30, 40, 50, 75, 100, and 150 mg. Analyses were performed at 6 h and pH 5. Each value is the mean of three independent experiments; standard deviation below 5%.

Based on the results found with MR-PDA, the MR-DPA removal capacity was evaluated by ICP-OES using solutions containing Cu^2+^ (37 ppm), Pb^2+^ (25 ppm), and Ag^+^ (28 ppm) at pH 5 to avoid the interference of Fe^3+^. At pH 5, the cations solution presented high turbidity, probably due to the formation of colloids or non-soluble particles (Figure 10). The solution becomes clear and colorless after adding 30 mg of MR-DPA to the mixture. Kołodynska (2011) has described a comparable phenomenon in systems containing Cu^2+^ ions [38]. The stability constants of the complexes formed with the CR prevent or reverse the occurrence of precipitation. The ICP-OES showed that MR-DPA had a 94% retention of Cu^2+^ at 100 mg (3.5 mg Cu^2+^/g), a 98% retention of Pb^2+^ at 40 mg (8.2 mg Pb^2+^/g), and a 98% retention of Ag^+^ at 30 mg (9.1 mg Ag^+^/g), respectively. It is important to mention that MR cannot remove Fe^3+^. Upon comparing the results obtained from the MR (150 mg: Pb^2+^ 26%, Ag^+^ 42%) to those achieved through MR-DPA for Pb^2+^ (40 mg: 98%) and Ag^+^ (30 mg: 91%), it is evident that the chelating molecule positively impacts the adsorption capacity of the CR (Table 1).

**Figure 11:**
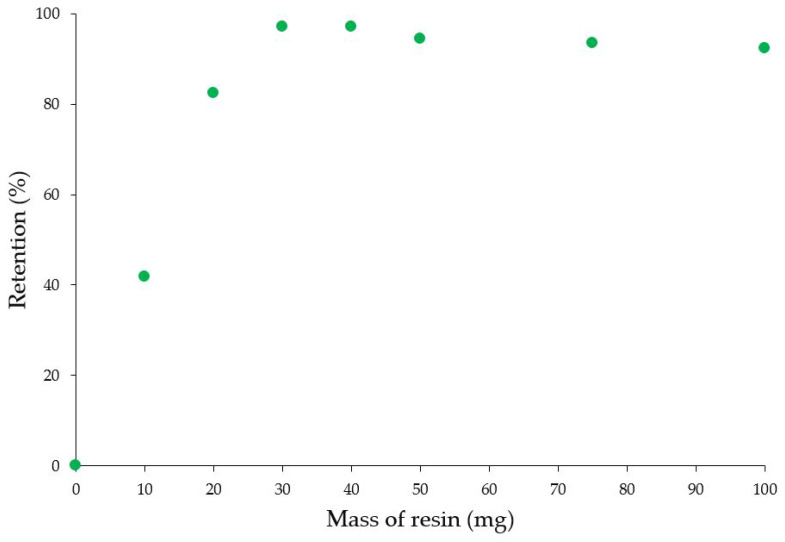
Effect of the MR-AMP dose on the Ag^+^ retention percentage in a modified batch test. Analyses were performed at 6 h and pH 2. Each value is the mean of three independent experiments; standard deviation below 5%.

The removal capacity of MR-AMP was evaluated by ICP-OES using solutions containing Cu^2+^ (36 ppm), Pb^2+^ (30 ppm), and Ag^+^ (53 ppm) at pH 3.4. After the treatment with MR-AMP, the analysis of the solutions revealed 97% of Ag^+^ retention from the solution using 30 mg of resin (17.1 mg Ag^+^/g) without removing any other metal cation under these experimental conditions (Figure 11, Table 1). This result shows the potential of MR-AMP as a selective dosimeter for Ag^+^. Additionally, a solution with Fe^3+^ was used for the MR-AMP column test.

Based on the findings, CR’s adsorption characteristics differ based on the chelating unit and metal ion involved. The interaction mechanism between the CR and the metal cations could resemble the means described for the Dowex M-4159 resin, which bears the bis(2-pyridyl methyl)amine [37,38]. Under specific pH values, the nitrogen atoms in PDA, DPA, and AMP can coordinate with metal cations. The pKa values for these compounds are as follows: PDA, 4.5; DPA, 0.8 and 4.8; AMP, 2.3 and 8.8. During the adsorption studies of MR-AMP, MR-PDA, and MR-DPA at pH values of 2, 3.6, and 5, respectively, sp^3^ nitrogen atoms may become partially protonated (except for DPA), as well as some sp^2^ nitrogen atoms, which may have the ability to coordinate with the metal ions. The mechanism could be described for MR-AMP and an M^2+^ as follows:

R-NH-CH_2_-*o*-Py + H+ ⇄ R-NH^+^-CH_2_-*o*-Py

R-NH^+^-CH_2_-*o*-Py + M^2+^ ⇄ R-NH^+^-CH_2_-*o*-Py→M^2+^

R-NH^+^-CH_2_-*o*-Py→M^2+^⇄ R-N-CH_2_-*o*-Py→M^2+^ + H^+^

or

R-[NH^+^-CH_2_-*o*-Py]_2_ + M^2+^ ⇄ R-[NH^+^-CH_2_-*o*-Py]_2_→M^2+^

R-[NH^+^-CH_2_-*o*-Py]_2_ + M^2+^ ⇄ [R-N-CH_2_-*o*-Py]_2_→M^2+^ + 2 H^+^

where R = resin matrix, and *o*-Py = Pyridine *o*-substituted

**Figure 12:**
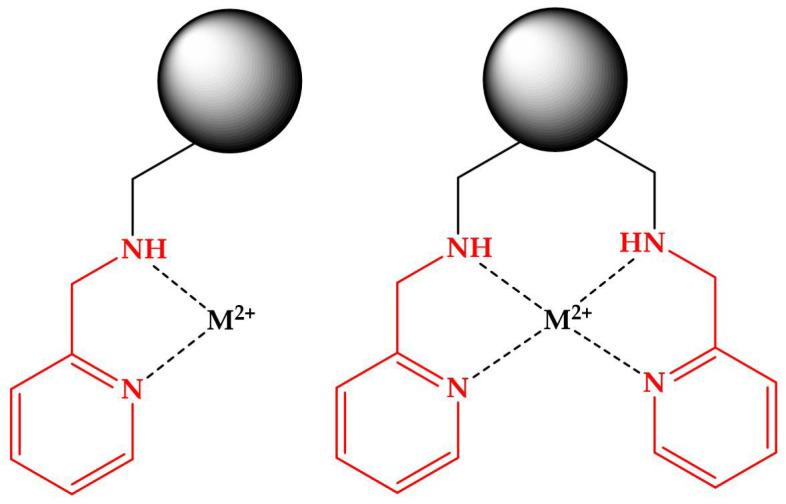
Proposed structure of the metal complexes formed in the MR-AMP.

The donor atom’s affinity to the metal ion determines the last equilibrium. Typically, the affinity is as follows: heavy M^2+^ ion >>> alkali metal ions > H^+^ [37]. The metal’s coordination sphere (Cu^2+^, pentacoordinate; Fe^3+^, octacoordinate; Pb^2+^ and Ag^+^, tetracoordinate) can be accomplished with the nitrate ion and water molecules. Figure 12 illustrates the potential structure of the complexes formed. The electrostatic interaction is also possible because of the protonated resin’s chelating unit, the metal ion, and the negatively charged counterion [38,39,40]. The formation of hydrogen bonds cannot be discarded. These possibilities may coexist at the same time, depending on the pH.

Notably, MR-PDA was the most efficient CR, as evidenced by 84% Ag^+^ cation adsorption at pH 7. This CR had high reusability and an average adsorption of over 74%. According to the study, MR-DPA-Ag^+^ has a higher adsorption capacity (18.4 mg/g) than Ghassabzadeh et al.’s reported adsorption capacity of 8.5 mmol/g. 

The MR-AMP demonstrated a reusability of three cycles. On the other hand, Martinez-Meza’s CR, which exhibited similar selectivity, was found to have no reusability. Moreover, only 50% of the cation was released after a 12 h treatment [27]. Martinez-Meza’s CR and MR-AMP showed a better adsorption capacity at a pH close to 2, which indicates that a higher acidity medium is more favorable for Fe^3+^ adsorption (Table 2).

Singh and Chahar synthesized a CR called THQSA that was highly selective for Cu^2+^ (pH = 5.9) and Fe^2+^ (pH = 3.9) [41]. It was found that MR-AMP retained 10% of the cation at pH 3, while the THQSA resin retained 87% at the same conditions. Ghassabzadeh et al. (2010) reported that expanded perlite has an adsorption capacity of 8.5 mg/g of Ag^+^ at pH = 6.5, while MR-AMP removed 17.1 mg/g of Ag^+^ at 3.4 (batch test) [26]. The difference could come from the influence of the binder on the cation-trapping mechanism [19]. However, the exposure time and pH influenced the cation adsorption capacity of MR-AMP and THQSA [40]. 

The variance in selectivity between the batch and flow methods may be due to resin swelling caused by water exposure. The flow method enables a more thorough analysis of selectivity by exposing the active sites of the CR.

As we can notice, the CRs MR-PDA and MR-DPA have the potential for environmental applications as wastewater treatment due to their simultaneous cation adsorption capacity. Meanwhile, the selective removal of Ag^+^ achieved by MR-AMP represents an alternative to Ag^+^ recovery in the mining industry from waste.

### 3.4. Chelating Resins Reusability

**Figure 13:**
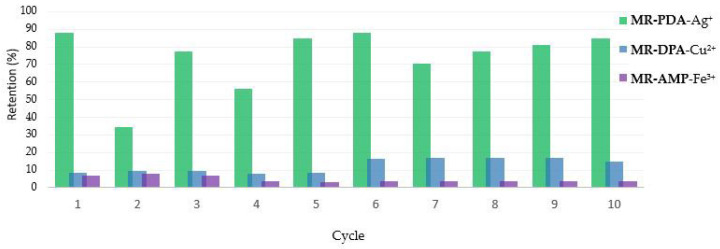
Determination of the number of adsorption–desorption cycles for each CR by a column test over ten cycles. Analyses were performed at 10 ppm, pH 4.3 for Ag^+^; 5.0 for Cu^2+^; and 2.4 for Fe^3+^. Rate 10 mL/min. UV-Vis 382 nm Ag^+^, 510 nm Cu^2+^, 240 nm Fe^3+^.

Reusability tests were performed on the metallic salt solution with the highest selectivity for each CR. A 10 ppm Ag^+^ cation solution was used at pH 4.3 and room temperature for MR-PDA. There was observed to be 88% retention in the first cycle (17.6 mg Ag^+^/g). However, the subsequent six cycles showed a fluctuation in percentage retention, followed by a steady decrease during the subsequent three cycles. These results suggest that MR-PDA has a reusability of 10 cycles with an average adsorption of 74% or 14.8 mg Ag^+^ /g (Figure 13).

The resin MR-DPA showed different behavior than MR-PDA. A 10 ppm Cu^2+^ cation solution at pH 5 was used at room temperature for MR-DPA. The percent recovery was between 8 and 10% until the 5th cycle, and from the 6th cycle onwards, this value doubled until the 10th cycle. The average recovery for the Cu^2+^ on MR-DPA was 8.7% (1.7 mg Cu^2+^/g), with five reusability cycles (Figure 13).

A 10 ppm Fe^3+^ cation solution at pH 2.4 and room temperature was used for MR-AMP. The results showed that the best percent recovery towards Fe^3+^ was observed in the second cycle. After the fourth cycle, the percent recovery decreased by half until the tenth cycle. The average percent recovery after ten cycles for MR-AMP was 4.7% (0.9 mg Fe^3+^/g), with a reusability of three cycles (Figure 13).

## 4. Conclusions

The three CRs synthesized are suitable adsorbents for Ag^+^, Cu^2+^, Pb^2+^, and Fe^3+^ cations due to their ability to capture various metal ions with differing affinities. The maximum metal adsorption value for Ag^+^ was 85% (18.4 mg/g) of MR-PDA at pH 7; for Cu^2+^, it was 94% (3.5 mg/g) of MR-DPA at pH 5; for Pb^2+^, it was 98% (8.2 mg/g) of MR-DPA at pH 5; and for Fe^3+^, it was 93% (29.8 mg/g) of MR-PDA. These values show the versatility of the CRs in metal removal.

MR-PDA was the most efficient resin, strongly favoring Ag^+^ and Fe^3+^. It can be reused multiple times. MR-DPA prefers Cu^2+^ and Pb^2+^, while MR-AMP has an affinity for Ag^+^. MR-PDA and MR-DPA have the potential for environmental applications in wastewater treatment due to their simultaneous cation adsorption capacity. Additionally, MR-AMP’s selective adsorption of Ag^+^ offers an alternative method for recovering Ag^+^.

Based on the experimental working conditions, the number of adsorption–desorption cycles, the mechanical stability, and the observed reproducibility, the CRs prepared herein offer an affordable, effective, and sustainable alternative for treating wastewater from mining operations, purifying drinking water, and recovering metals.

## Figures and Tables

**Table 1:** Maximum metal retention percentage varying the adsorbent dose of each CR after six hours (batch procedure).

Resin	Cation	Maximum Retention %	Adsorbent Dose (mg) for Maximum Retention
MR	Ag^+^ Pb^2+^ Fe^3+^	42 26 83	150 150 150
MR-PDA	Fe^3+^ Ag^+^ Pb^2+^	93 91 80	10 150 150
MR-DPA	Ag^+^ Pb^2+^ Cu^2+^	91 98 94	30 40 100
MR-AMP	Ag^+^	97	30

**Table 2:** Comparison of the adsorption properties of some reported CRs and MR-PDA, MR-DPA, and MR-AMP.

Adsorbent	Metals/Adsorption Capacity	pH	Reference
MR-AMP	Fe^3+^ (4.3 mg/g)	2	This work
Dowex-4195	Fe^3+^ (7.5 mg/g)	2	[27]
THQSA	Fe^2+^ (9.1 × 10^3^ mg/g)	2	[41]
MR-DPA	Cu^2+^ (2.2 mg/g)	5	This work
Bis-(phosphonomethyl)amine resin	Cu^2+^ (569.5 mg/g)	5	[42]
Polychloromethylestyrene resin	Cu^2+^ (161.9 mg/g)	5	[28]
Poly(6-acryloyl amino-*N*-hydroxyhexanamide) resin	Cu^2+^ (238.6 mg/g)	5	[29]
THQSA	Cu^2+^ (10.6 × 10^3^ mg/g)	5	[41]
Expanded perlite	Cu^2+^ (2.0 mg/g)	6.5	[26]
Morin and Merrifield resin	Cu^2+^ (19 × 10^−6^ mg/g)	7	[25]
MR-PDA	Ag^+^ (18.4 mg/g)	7	This work
Expanded perlite	Ag^+^ (8.5 mg/g)	6.5	[26]

## Data Availability

The data presented in this study are available on request from the corresponding author.

## Supplementary Materials

The following supporting information can be downloaded at: https://www.mdpi.com/article/10.3390/polym15132778/s1, Figure S1: Calibration plots of Ag+, Cu2+, and Fe3+ by UV-Vis at 281, 240, and 510 nm, respectively; Figure S2: Determination of cation retention percentage of chelating resins by flow procedure; Table S1: Cation retention by chelating resins (flow procedure); Table S2: Data on the reusability of chelating resins were obtained through analysis using the flow procedure.
