# Supplementary material for: Efficient and Sustainable Bidentate Amines-Functionalized Resins for Removing Ag+, Cu2+, Pb2+, and Fe3+ from Water
Source: Polymers (Basel). 2023 Jun 22;15(13):2778. doi: 10.3390/polym15132778 (PMC10346304; doi:10.3390/polym15132778)
Supplement: Supplementary file 1 [file polymers-15-02778-s001.zip › polymers-2422117-supplementary.pdf]

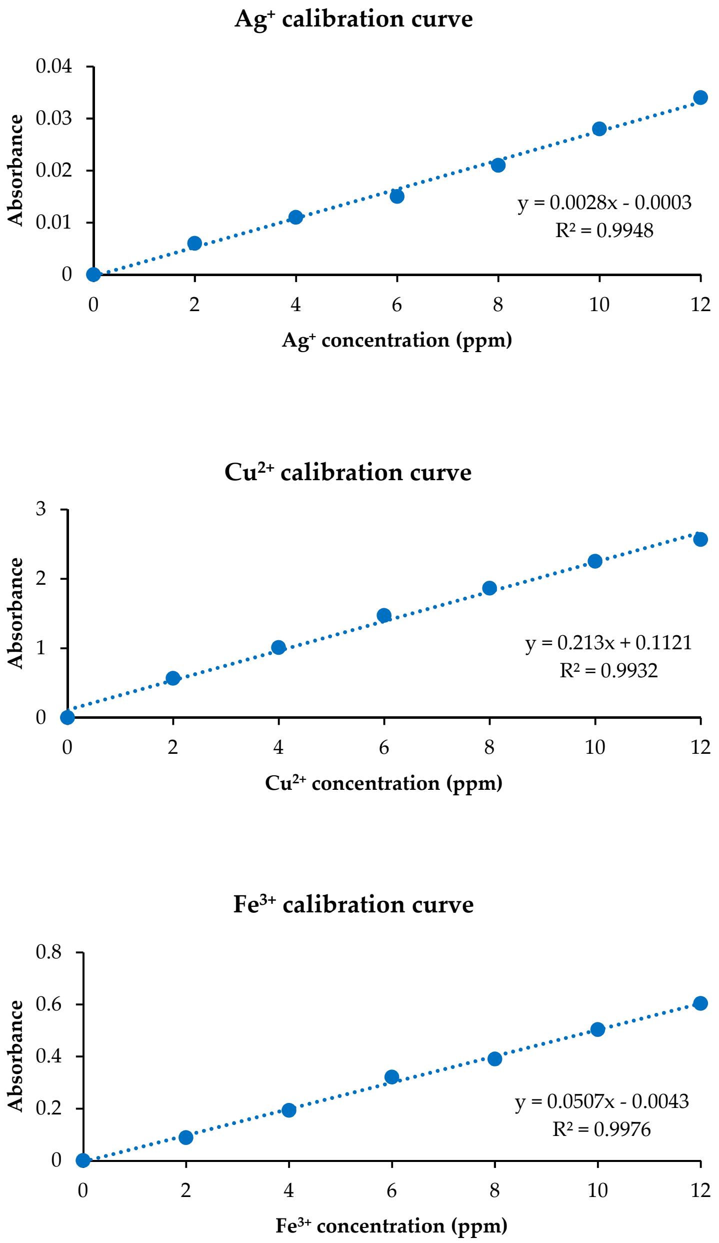

Figure S1. Calibration plots of Ag^{+}, Cu^{2+}, and Fe^{3+} by UV-Vis at 281, 240, and 510 nm, respectively.

Table S1. Cation retention by chelating resins (flow procedure).

**MR-PDA**-Ag^+ (A_i=0.028, λ=382 nm; C_i = 10 ppm)				
**pH**	**vel=10 mL/min**		**vel=5 mL/min**	
	A_f	C_f (ppm)	A_f	C_f (ppm)
7	0.004	1.535	0.002	0.821
6	0.006	2.250	0.005	1.892
5	0.015	5.464	0.028	10.000
4	0.024	8.678	0.028	10.000

A_i: initial absorbance; A_f: final absorbance; C_i: initial concentration; C_f: final concentration

**MR-DPA**-Cu^{2+} (A_i=2.253, λ=240 nm; C_i = 10 ppm)				
**pH**	**vel=10 mL/min**		**vel=5 mL/min**	
	A_f	C_f (ppm)	A_f	C_f (ppm)
5	2.003	8.877	2.031	9.008
4	2.253	10.000	2.253	10.000
3	2.075	9.215	2.101	9.337
2	2.197	9.780	2.218	9.886

A_i: initial absorbance; A_f: final absorbance; C_i: initial concentration; C_f: final concentration

**MR-AMP--**Fe^{3+} (A_i=0.503, λ=510 nm; C_i = 10 ppm)				
**pH**	**vel=10 mL/min**		**vel=5 mL/min**	
	A_f	C_f (ppm)	A_f	C_f (ppm)
3	0.481	9.402	0.449	8.940
2	0.393	7.836	0.393	7.737

A_i: initial absorbance; A_f: final absorbance; C_i: initial concentration; C_f: final concentration

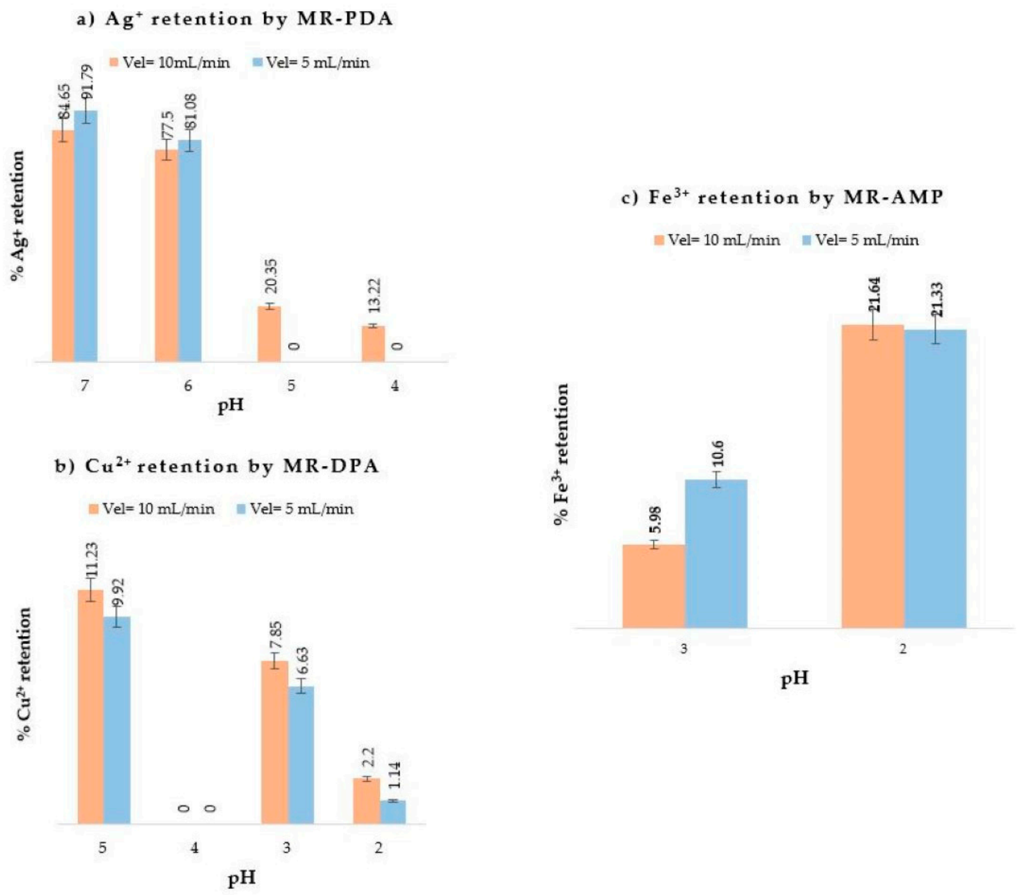

Figure S2. Determination of cation retention percentage of chelating resins by flow procedure. a) **MR-PDA**-Ag^{+}. b) **MR-DPA**-Cu^{2+}. c) **MR-AMP**-Fe^{3+}. At a flow rate of 10 mL/min, the blue bar displays data for a 10 ppm solution, while the orange bar represents the same concentration at a flow rate of 5 mL/min. The error bars have a 95% confidence level.

Table S2. Data on the reusability of chelating resins were obtained through analysis using the flow procedure.

**MR-PDA**-Ag^{+} (**A_i**=0.028, λ=382 nm; **C_i** = 10 ppm)					
**vel=10 mL/min**					
**Cycle**	**A_f**	**C_f** (ppm)	**Cycle**	**A_f**	**C_f** (ppm)
1	0.003	1.178	6	0.003	1.178
2	0.018	6.535	7	0.008	2.964
3	0.006	2.250	8	0.006	2.250
4	0.012	4.392	9	0.005	1.892
5	0.004	1.535	10	0.004	1.535

A_i: initial absorbance; A_f: final absorbance; C_i: initial concentration; C_f: final concentration

**MR-DPA**-Cu^{2+} (**A_i**=2.253, λ=240 nm; **C_i** = 10 ppm)					
**vel=10 mL/min**					
**Cycle**	**A_f**	**C_f** (ppm)	**Cycle**	**A_f**	**C_f** (ppm)
1	2.058	9.135	6	1.898	8.384
2	2.043	9.065	7	1.883	8.314
3	2.035	9.027	8	1.879	8.295
4	2.075	9.215	9	1.876	8.281
5	2.063	9.159	10	1.921	8.492

A_i: initial absorbance; A_f: final absorbance; C_i: initial concentration; C_f: final concentration

**MR-AMP**-Fe^{3+} (**A_i**=0.503, λ=510 nm; **C_i** = 10 ppm)					
**vel=10 mL/min**					
**Cycle**	**A_f**	**C_f** (ppm)	**Cycle**	**A_f**	**C_f** (ppm)
1	0.148	3.003	6	0.317	6.337
2	0.092	1.899	7	0.328	6.554
3	0.160	3.240	8	0.309	6.179
4	0.305	6.100	9	0.315	6.297
5	0.332	6.633	10	0.321	6.416

A_i: initial absorbance; A_f: final absorbance; C_i: initial concentration; C_f: final concentration
